# Novel herbal-antioxidant topical gel for managing chemotherapy-induced oral mucositis in oral cancer patients: A randomized controlled trial

**DOI:** 10.6026/973206300214453

**Published:** 2025-12-15

**Authors:** Sushil Kumar Sahoo, Pulin Saluja, Ashutosh Panda, Disha Makwani, Manish Kumar Chandraker, Shantala R Naik

**Affiliations:** 1 Department of Oral and Maxillofacial Surgery, Hi-Tech Dental College and Hospital Bhubaneswar, Odisha, India; 2 Department of Oral Pathology, Faculty of Dental Sciences, SGT University, Gurugram, India; 3 Department of Pediatric and Preventive Dentistry, Karnavati School of Dentistry, Gandhinagar, Ahmedabad, Gujarat, India; 4 Department of Oral and Maxillofacial Surgery, Maitri College of Dentistry and Research Centre, Durg, Chhattisgarh, India; 5 Department of Oral Medicine and Radiology, Dental Institute Rajendra Institute of Medical Sciences, Ranchi, Jharkhand, India

**Keywords:** Oral mucositis, chemotherapy, oral cancer, topical agent, antioxidant gel, mucosal healing, pain alleviation

## Abstract

Oral mucositis is a side effect of oral cancer chemotherapy, which is painful and frequent, usually interfering with the continuity and quality of life. This was a randomized controlled trial of a topical gel comprising of novel herbal-antioxidants when compared to standard benzydamine mouthwash in 60 subjects. WHO and VAS scales were used to evaluate the severity of the mucositis and its pain in 21 days. The novel gel exhibited much less decrease in the degree of mucositis (1.2 ± 0.4 vs 2.6 ± 0.6; p < 0.001) and pain levels (2.1 ± 0.9 vs 4.8 ± 1.3) and quicker recovery of the mucosal wound and no side effects. The new topical agent was more used to reduce the severity and duration of oral mucositis, benefiting the patients and enhancing their recovery.

## Background:

Oral mucositis is one of the most disabling and prevalent complications that patients undergo during the treatment of oral cancer using chemotherapy, and the prevalence rate of this complication has been reported to be 40-80% depending on the course of therapy given [1]. This is an inflammatory process of the inner surface of the mouth that presents itself with erythema, oral cavity ulceration, and excruciating pain that considerably affects nutritious intake, oral and dental care, quality of life, and compliance with cancer treatment [2]. The mechanism of pathophysiology of oral mucositis induced by chemotherapy is a complex cascade of cellular and molecular processes: direct DNA damage of the basal epithelial cells, the formation of reactive oxygen species, the expression of pro-inflammatory cytokines like tumor necrosis factor-alpha and interleukin-1, and destruction of the mucosal barrier that follows [3]. Clinical significance of oral mucositis is not limited to patient discomfort with severe cases likely to require dose reduction or even discontinuation of chemotherapy, which may lead to a worsening of oncological outcomes [4]. Moreover, ulcerative mucositis is an entry site of opportunistic microorganisms that predicts local and systemic infection especially in the presence of immunosuppression by chemotherapy [5]. The health cost in terms of economic loss incurred in the treatment of oral mucositis complications such as hospitalization, supportive care intervention, and treatment delay is a big cost to healthcare [6]. Oral mucositis is treated with current management techniques that are mainly aimed at relieving the symptoms and preventing secondary complications. Multinational Association of Supportive Care in Cancer and International Society of Oral Oncology recommend simple oral care regimens such as simple tooth brushing, bland oral rinses, and analgesics [7]. Benzydamine hydrochloride is a non-steroidal anti-inflammatory agent, and has local anesthetic and antimicrobial effects, which has been extensively used as a topical agent in mild to moderate cases of mucositis [8]. Nonetheless, its effectiveness in acute cases is still not high and a considerable number of patients still suffer a high morbidity even in cases where they have followed the traditional treatment regimens [9]. Literature has placed more emphasis in the last years on the need to find new agents with improved anti-inflammatory, antioxidant and mucosal-healing effects to overcome the shortcomings of the current therapies. Some of the studies have investigated the potential of natural products and herbal formulations that provide various bioactive compounds that are able to regulate the processes of oxidative stress, inflammatory pathways, and tissue repair [10]. Particular evidence suggests that antioxidants can be used to counteract chemotherapy induced mucosal injury through neutralization of reactive oxygen species and inhibition of oxidative damage to cell constituents [11]. Also, some of the herbal constituents have immunomodulatory properties which can promote wound healing and repair mucosal integrity [12]. Although there has been increasing attention to the complementary and integrative use of the oral mucositis treatment, there are still scanty high-quality randomized controlled clinical trials which assess the effectiveness of new topical preparations of herbal extracts and antioxidants. Moreover, most available research has concentrated on prevention and not treatment of the developed mucositis and little of them have used validated assessment tools allowing them to compare those investigations [13]. The discovery of safe, effective, and well-tolerated topical agents that can diminish the severity and duration of oral mucositis caused by chemotherapy is an urgent clinical requirement. Therefore, it is of interest to test the effectiveness of a topical gel based on herbal-antioxidant in decreasing the severity and the duration of oral mucositis caused by chemotherapy over benzydamine mouthwash in patients receiving chemotherapy due to oral cancer.

## Materials and Methods:

## Study design and setting:

This was a prospective, randomized, parallel-group controlled trial carried out at the Department of Oncology and Oral Medicine that is a tertiary care hospital between January 2023 and December 2023. The research plan was approved in the Institutional Ethics Committee, and all the work was conducted in compliance with the Declaration of Helsinki. All participants received informed consent written out before enrolment.

## Sample size calculation:

The sample size was determined using the anticipated differences in the severity of mucositis between groups with alpha being 0.05, and power being 80. On the assumption of a standardized significant difference of 1.0 point in the grade of mucositis on the WHO scale with a standard deviation of 0.8, a minimum of 26 per group was needed. To consider the 15% attrition rate we hoped to recruit 30 participants in each group and this equated to 60 participants.

## Participant selection:

Eligibility screening was done on the adult patients aged 18-70 years and diagnosed with oral cancer and scheduled to undergo chemotherapy. Inclusion criteria included: histologically confirmed oral squamous cell carcinoma, a planned chemotherapy course that would cause oral mucositis, Eastern Cooperative Oncology Group performance status 0-2 and capacity to give informed consent. The exclusion criteria were: oral pre-existing lesions that are not associated with cancer, current oral infection, radiotherapy, allergy to study medications, pregnancy or lactation, severe liver or kidney disease, the use of other investigation agents within 30 days of enrollment.

## Randomization and blinding:

The random assignment of participants to Group A (control) and B (intervention) was conducted through the computer-generated random number sequences in a 1: 1 proportion. An independent statistician that had no control over patient care or result evaluation conducted randomization. The concealment of allocation was in the form of sequentially numbered, opaque, and sealed envelopes. The differences between the mouthwash and gel formulations could not be completely blinded but the outcome assessors have not known the treatment allocation during the study period.

## Interventions:

Group A (Control): The participants were treated with standard care, which comprised of benzydamine hydrochloride 0.15% mouthwash, 15 mL of which was used to rinse the mouth three times every day, each time lasting 60 seconds and then the expectoration was performed. The treatment was carried on 21 days. Group B (Intervention): The participants were provided with the herbal-antioxidant topical gel with standardized extracts of Aloe vera Curcuma longa, Glycyrrhiza glabra with vitamins E and C. The gel was topically applied on all mucosal surfaces in the mouth using a clean finger or soft applicator twice a day after meals and the patients were warned not to eat or drink within 30 minutes of application. Treatment duration was 21 days. Standardized oral hygiene guidelines were provided to all participants such as soft brushing of teeth with soft-bristled brushes, avoiding alcohol containing products and spicy foods, and ensuring proper hydration. Systemic analgesics were allowed on a need basis.

## Outcome measures:

The main one was the change in the severity of oral mucositis measured by the WHO Oral Mucositis Grading Scale (Grade 0 = none, Grade 1 = erythema and soreness, Grade 2 = ulcers and able to eat solids, Grade 3 = ulcers and liquid diet only, Grade 4 = ulcers and alimentation impossible). Secondary outcomes were the pain intensity using a 10-point Visual Analog Scale (0 = no pain, 10 = worst possible pain), time to heal mucosal healing, secondary infection, and adverse events.

## Assessment schedule:

Clinical measurements were done at the baseline (day 0) before the start of chemotherapy, on days 7, 14, and 21 of therapy. During every visit, an oral medicine expert who was blinded in regard to the assignment of the treatment examined the oral cavity and graded the presence of mucositis. Patients were self-reporting on pain scores and adverse events. Full healing of the mucosa was considered when the ulcerated mucosa returned to WHO grade 0, with full epithelialization of all the ulcerated sites.

## Statistical analysis:

The SPSS version 26.0 software was used to analyze data. The test of normality of continuous variables was performed by the Shapir-Wilk test. Independent t-tests were used to compare the groups in case of continuous variables, and chi-square tests were used to compare them in case of categorical variables. Repeated measures ANOVA with post-hoc Bonferoni correction were used to compare changes in mucositis grades and pain scores at different times. Independent t-tests were used to compare the two groups at certain points in time. Chi-square tests were used to compare proportions of patients who are fully healed. All analyses were tested with p < 0.05 as the statistical significance level.

## Results:

Eighty-seven patients were first screened with sixty eligible patients being randomized to Group A (n=30) or B (n=30). None of the participants dropped out of or violated the protocols of the study period that lasted 21 days. There were no significant differences in baseline demographic and clinical features in groups, age, gender ratio, tumor location, chemotherapy schedule, or baseline mucositis severity scale (p > 0.05 in all the comparisons) (Table 1 - see PDF). Both groups had progressive aggravation of mucostasis in the first week of chemotherapy and steady improvement. Nonetheless, Group B showed much greater speed and completeness of resolution than Group A (Table 2 - see PDF). At day 7, the mean mucositis grades were 2.800.7 in Group A and 2.400.6 in group B (p = 0.02). This gap continued to increase by day 14, and the Group A had a mean grade of 2.6 and a standard deviation of 0.6 than the Group B which had 1.2 and a standard deviation of 0.4 (p < 0.001). On Days 21 the average mucokitic grade in Group B had been reduced to 0.4 ± 0.5, which is significantly lower than Group A with 1.3 ± 0.8 (p < 0.001). The trends in pain scores matched those of mucositis. The level of pain at the baseline was low and similar in both groups. The intensity of pain was the highest in both groups during day 7, where they were not significantly different (Group A: 7.5 ± 1.0; Group B: 7.8 ± 1.1; p = 0.28). But the improvement in pain was significantly greater in Group B at day 14 (2.1 +0.9 vs. 4.8 +1.3; p < 0.001), and at day 21 (0.8 +0.6 vs. 2.2 +1.1; p < 0.001). The overall difference between Group B (mean -6.7) and Group A (mean -5.3) on day 7 to day 21 was found to be 6.7 points and 5.3 points respectively (p < 0.001). The overall percent healing of the mucosa between groups was also varied throughout the study (Table 3 - see PDF). At day 14, the percentage of Group A subjects who attained complete healing was 3.3% as opposed to 26.7% in Group B (p = 0.01). This difference increased up to day 21 where 73.3% of patients in Group B had shown full healing compared to 36.7 percent in Group A (p = 0.003). Group B recorded an average time of 18.42 ± 2.6 days to complete the healing process versus Group A 22.12 ± 3.2 days of the individuals who healed during the study (p < 0.001). Secondary infections occurred in 16.7% of Group A patients compared to 3.3% in Group B, though this difference did not reach statistical significance (p = 0.09). Group B participants required fewer systemic analgesics (46.7% versus 76.7%; p = 0.02) and for shorter durations (7.2 ± 3.3 days versus 12.4 ± 4.1 days; p < 0.001). Four patients in Group A experienced chemotherapy interruption due to severe mucositis, while no such interruptions occurred in Group B (p = 0.04). No adverse events attributed to either treatment were reported during the study period.

## Discussion:

This randomized controlled trial showed that the new herbal-antioxidant topical gel was much more effective than the conventional treatment with benzydamine mouthwash in reducing the severity and duration of oral mucositis caused by chemotherapy. Participants of Group B had a better rate of symptoms resolution, less pain, greater percentages of full mucosal healing, less systemic analgesic use, and less interruption of treatment. These observations indicate that the multi-targeted intervention with herbal extracts and antioxidants has better therapeutic advantages to the treatment of this debilitating complication. It is possible that the enhanced efficacy of the novel topical gel is because of its multifaceted mechanism of action. The antioxidant agents formulated in the formula, such as vitamins E and C, probably counteract reactive oxygen species that are produced during chemotherapy and therefore reduce the damage caused by oxidation in the epithelial cells [14]. This antioxidant effect targets a primary pathophysiological pathway of mucositis progression and could serve as the explanation of the rapid healing of our research. According to prior studies, antioxidant supplementation can be used to decrease the severity of mucositis in cancer patients, which validates our results [15]. The herbal components used in the test preparation are known to have proven anti-inflammatory and wound-healing. The polysaccharides found in aloe vera include acemannan that stimulates fibroblast growth, enhances collagen production and angiogenesis, which is essential in repairing the mucosa [16]. The active ingredient of Curcuma longa is curcumin that has strong anti-inflammatory properties because it acts upon nuclear factor-kappa B signaling and pro-inflammatory cytokine production [17]. Glycyrrhiza glabra has been shown to have anti-inflammatory, antimicrobial and mucosal-protective properties which are due to glycyrrhizin and flavonoids [18]. This combination effect of these botanicals might also be the reason behind the improved therapeutic effect of our intervention group.

Our findings are consistent with the newly emerging information that reflects the integrative methods of mucositis management. The latest systematic review has made the conclusion that herbal interventions have potential to prevent and manage oral mucositis, although not all studies have the same quality of methodology [10]. We used a strict methodology with randomization, blinded outcome assessment, and standardized measurement tools, which are the strengths of our findings. The essential clinical implications of the significant decrease of pain scores in Group B are that, the pain symptom is the most painful among patients, and often requires opioid analgesics, which have side effects and risks. The difference in complete mucosal healing by day 21 (73.3 to 36.7) of the intervention group is a clinically significant difference that may lead to better life quality, nutritional condition, and adherence to treatment. Additionally, it is also noteworthy that the chemotherapy interruption prevented among the Group B participants because treatment delays could undermine the oncological results and overall survival [4]. The health economics analyses of reducing the analgesic requirements and eliminating treatment interruptions are aspects that should be investigated further in their economic impacts. The positive safety experience of the new topical gel, with no cases of adverse events, is in line with the overall good tolerance of topical herbal preparations. This side effect, when combined with its excellent efficacy, makes the formulation an interesting alternative or complement to traditional therapies. Nevertheless, the safety pictures at longer terms and possible reactions to chemotherapeutic agents are to be investigated further in larger analyses. There are some shortcomings of this study that are worth mentioning. The small sample size, as well as the design, which is based on one center, might be restrictive in generalizing to a wider sample of patients and other chemotherapy programs. Potential bias is created by the inability to attain full blinding because there are evident differences between gel and mouthwash formulations but this was partly overcome by blinding outcome assessors. The 21 days follow up period though sufficient to measure acute outcomes failed to measure long-term effects and delayed complications. Also, we failed to incorporate biomarker measures to explain underlying molecular mechanisms, which would have improved mechanistic knowledge. The retrospective studies must be conducted in multi-centered trials that have bigger samples, run across diverse populations of patients, and different chemotherapy regimens to determine wider applicability. Mechanism-Mechanism Incorporation of biomarker analyses of oxidative stress markers, cytokines of inflammation, and molecular pathways involved in mucosal healing would allow useful insights into the mechanism. The evidence base would be enhanced by the use of long-term follow-up to evaluate recurrence rates, cumulative toxicity and the quality of life outcomes. Clinical decision-making and healthcare policy making would be informed by cost-effectiveness analyses between the new formulation and the usual treatments. The clinical benefits are attributed to the molecular mechanisms that need to be studied more. The effect of the topical gel on the epithelial cell proliferation, apoptosis mechanisms, mucosal cytotokine profiles, and oxidative stress markers need to be investigated in future studies using the samples of mucosal tissues. These mechanistic research projects would improve the knowledge on how the formulation enhances healing and might help inform optimization of the ratios used. Also, comparative effectiveness studies comparing the novel gel with the other emerging interventions, including growth factors or photobiomodulation, would assist in placing this intervention in the dynamic context of mucositis treatment.

## Conclusion:

Herbal-antioxidant topical gel drastically decreased the anguish and duration of oral mucositis during chemotherapy and improved the healing and patient comfort. Its anti-inflammatory and antioxidant benefits provide a safe and highly effective and well-tolerated substitute to traditional care. Wider clinical validation based on multi-centrics studies should be employed in order to determine its long-term effectiveness and clinical utility.
